# Novel Roles of SH2 and SH3 Domains in Lipid Binding

**DOI:** 10.3390/cells10051191

**Published:** 2021-05-13

**Authors:** Szabolcs Sipeki, Kitti Koprivanacz, Tamás Takács, Anita Kurilla, Loretta László, Virag Vas, László Buday

**Affiliations:** 1 Department of Molecular Biology, Institute of Biochemistry and Molecular Biology, Semmelweis University Medical School, 1094 Budapest, Hungary; sipeki.szabolcs@med.semmelweis-univ.hu; 2 Institute of Enzymology, Research Centre for Natural Sciences, 1117 Budapest, Hungary; koprivanacz.kitti@ttk.hu (K.K.); takacs.tamas@ttk.hu (T.T.); kurilla.anita@ttk.hu (A.K.); laszlo.loretta@ttk.hu (L.L.); vas.virag@ttk.hu (V.V.)

**Keywords:** SH2 domain, SH3 domain, Src, protein tyrosine kinase, proline-rich sequences, lipid binding

## Abstract

Signal transduction, the ability of cells to perceive information from the surroundings and alter behavior in response, is an essential property of life. Studies on tyrosine kinase action fundamentally changed our concept of cellular regulation. The induced assembly of subcellular hubs via the recognition of local protein or lipid modifications by modular protein interactions is now a central paradigm in signaling. Such molecular interactions are mediated by specific protein interaction domains. The first such domain identified was the SH2 domain, which was postulated to be a reader capable of finding and binding protein partners displaying phosphorylated tyrosine side chains. The SH3 domain was found to be involved in the formation of stable protein sub-complexes by constitutively attaching to proline-rich surfaces on its binding partners. The SH2 and SH3 domains have thus served as the prototypes for a diverse collection of interaction domains that recognize not only proteins but also lipids, nucleic acids, and small molecules. It has also been found that particular SH2 and SH3 domains themselves might also bind to and rely on lipids to modulate complex assembly. Some lipid-binding properties of SH2 and SH3 domains are reviewed here.

## 1. Introduction

Every cell must process information from its environment and appropriately respond to any changes. An understanding the mechanisms that make this possible has long been a goal of biological research. In the three decades since the first reports that modular protein-binding domains interact with plasma membrane receptors and their downstream effectors, it has been established that such domains are involved in almost every corner of signaling in eukaryotes [1]. They allow signaling proteins to assemble into dynamic multiprotein complexes and provide mechanisms to translate one type of intracellular information into other types. For example, they help to convert enzyme-catalyzed chemical modifications of proteins and lipids into alterations in protein–protein interactions and protein subcellular localization [1]. They also drive evolution by providing ways to add new capabilities and connections to existing signaling networks [1].

Sadowski et al. first described a conserved, noncatalytic domain in cytoplasmic protein tyrosine kinases (PTKs), which comprises approximately 100 amino acid residues [2]. Analysis of the v-Fps/Fes cytoplasmic tyrosine kinase identified a region N-terminal to the kinase domain that, while not required for catalytic activity, was involved in the modification of the kinase activity and substrate recognition and was necessary for cellular transformation [2]. This element was termed the Src homology 2 (SH2) domain because this functionally important stretch of amino acids is conserved in Src and Abl tyrosine kinases and similarly positioned next to the kinase (SH1) domain [2]. The SH2 domain was defined as a reader responsible for the regulated assembly of signaling complexes triggered by tyrosine kinases because it binds to tyrosine-phosphorylated partner proteins in a phosphorylation-dependent and sequence-specific manner [3,4,5,6,7].

The cloning of the viral Crk oncogene, which encodes a small nonenzyme protein containing an SH2 domain, revealed a second noncatalytic element, which is also found in Src and Abl kinases [8]. The Src-homology domain 3 (SH3 domain) comprises approximately 60 amino acids and has a key role in signaling [8,9,10]. Once it became clear that the SH2 domain is a modular protein-binding domain, it was logical to investigate whether the SH3 domain had similar roles. Several SH3-binding proteins were identified in a screen using a purified SH3 domain with an expression library [10,11]. The fact that these were obviously signaling proteins suggested that the binding interactions were purposeful [12]. The consensus sites of SH3 attachment were narrowed down to short proline-rich motifs with a PXXP (where X is any amino acid) sequence at their centers [12]. The SH3 domain was the first example of a group of modular protein-binding domains that do not depend on post-translational modifications [13,14]. Such domains act constitutively to facilitate the assembly of dynamic complexes in various contexts. The individual interactions of constitutive binding domains tend to be weak; however, multiple domains can act together to mediate stronger and more stable interactions. Consistent with this concept, many proteins contain several SH3 domains, up to five in some cases [15,16].

The assembly of signaling complexes in different pathways usually occurs at the plasma membrane. Cell membranes are composed of a large number of different lipid molecules with variations in their head group and acyl chain structures. Alterations in local lipid composition also represent information that can be deciphered by lipid-binding domains [17]. Lipid-binding domain research was born with the discovery of protein kinase C (PKC), which led to the identification of conserved regions shared by specific PKC isoforms, now termed C1 and C2 domains, which display specific lipid-binding properties [18,19,20]. Later, a substrate of PKC called pleckstrin was shown to contain a phosphoinositide-binding region which was termed the pleckstrin homology (PH) domain [21]. Studies of structural data on C1, C2, and PH domains eventually led to the identification of additional lipid-binding domains. These individual domains were thereafter scrutinized to understand how they can bind lipids and membranes, translocate to membrane docking sites in the cell, and function to regulate the proteins which bear them. The importance of lipid–protein interactions in signaling is now well established [22].

The distinct protein-binding domains contain consensus amino acid sequences and, in many cases, specific secondary and occasionally tertiary structures. The individual domains within a span of amino acids in specific proteins are thus postulated on the basis of their homologies to consensus amino acid sequences and their folds. On the other hand, there are always disparities in the sequences that might allow further specificity. Moreover, these differences may result in the gain of a new binding ability towards additional ligands or the exchange of the originally defined binding target for others. Nevertheless, there is still a paucity of predictive data for protein-binding domains, meaning that they must be studied in vitro and in cells on an individual basis to characterize their functions. Interestingly, many SH2 domains have been shown to bind lipids in addition to phosphorylated tyrosine motifs. Conversely, some characterized SH3 domains do not recognize the canonical PXXP sequence, but instead bind to other protein motifs or ultimately stick to lipids. (Table 1) The lipid-binding properties of the SH2 and SH3 domains are the focus of this review.

## 2. Many SH2 Domains Themselves Browse Membrane Lipids besides Tyrosine Phosphorylated Proteins to Find the Matching Partners

Since its discovery, the approximately 100 amino acid-long SH2 domain has been identified in a wide variety of proteins in different eukaryotes, but primarily in metazoans. The human genome encodes 111 different SH2 proteins, including kinases, phosphatases, adaptors, and other signaling molecules with 121 SH2 domains altogether [23]. Structural analysis of a spectrum of SH2 domains and their complexes with tyrosine phosphorylated peptides revealed that SH2 domains have a cassette-like design made of antiparallel β strands flanked by two α helices [24]. Via a cationic binding pocket and a secondary binding site they specifically recognize a phosphorylated tyrosine and a few residues immediately C-terminal to it, respectively [23]. Quantitative analysis showed that SH2 domains bind phosphotyrosine-containing peptides casually with variable affinity [25,26]. Thus, the accuracy of specific protein interactions to support high-fidelity tyrosine kinase signaling must be enhanced by other mechanisms, such as protein compartmentalization, multi-protein complex formation, or phosphotyrosine-independent secondary protein interactions [27,28,29,30,31]. Membrane lipids were suggested to play a role in modulating cellular protein–protein interactions mediated by PTB or PDZ domains [32,33,34,35,36,37]. Early on, a small number of SH2 domains, including those of PI3K and PLC-γ, were reported to bind lipids, either inhibiting or increasing the activity of their parent proteins; however, until recently, the mechanisms, physiological significance, and universality of these findings have remained controversial [38,39,40,41].

The Abl tyrosine kinase SH2 domain was one of the first scrutinized in terms of lipid binding [42]. Abl is encoded by a proto-oncogene and is implicated in the regulation of diverse cellular functions [43]. It has a casual role in transformation and leukemia [44]. Abl is recruited to various subcellular locations, such as the nucleus, cytosol and plasma membrane, suggesting a complex cellular targeting mechanism. Tokonzaba at al. have reported that phosphatidylinositol-4,5-bisphosphate interacts with the Abl SH2 domain via an electrostatic mechanism on a site that overlaps with the phosphotyrosine-binding pocket [42]. According to their observations, R152 in the FLVRES motif is necessary for both phosphotyrosine recognition and for localization to phosphatidylinositol-4,5-bisphosphate-containing liposomes, while R175 is required for phosphoinositide binding. Here, it was speculated that phosphatidylinositol-4,5-bisphosphate interacts with the Abl SH2 domain primarily in the absence of tyrosine phosphorylation of its protein ligands.

**Figure 1:**
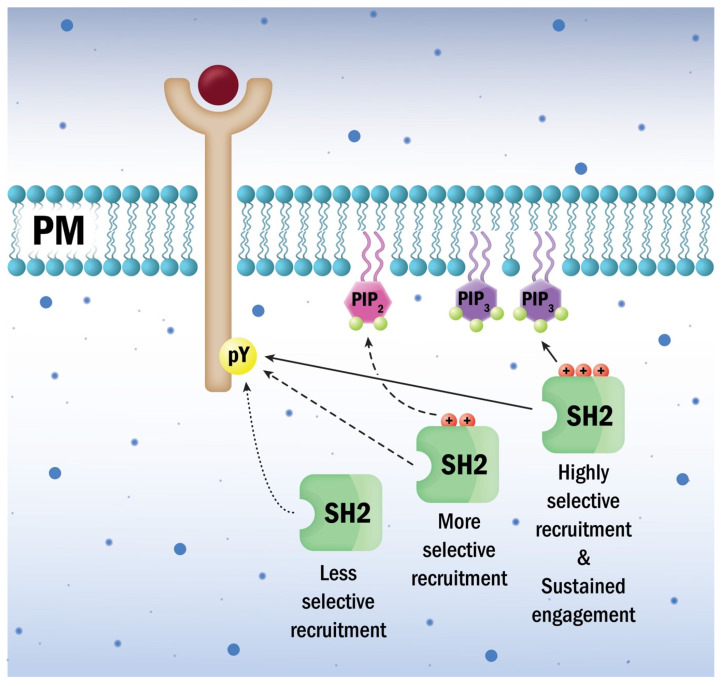
Lipid-binding abilities of some SH2 domains may contribute to spatio-temporal coordination of tyrosine kinase signaling hub dynamics. Alternative cationic patches distinct from the phosphotyrosine-binding pockets serve as the primary binding site for membrane lipids in most SH2 domains attracted to lipids. Phosphatidylinositol-4,5-bisphosphate or phosphatidylinositol-3,4,5-trisphosphate are preferred over other phosphoinositides for binding by these elements. The shape and net charge of the respective alternative cationic patches of specific SH2 domains provide further lipid recognition bias and may guide their host proteins to distinct membrane regions.

**Figure 2:**
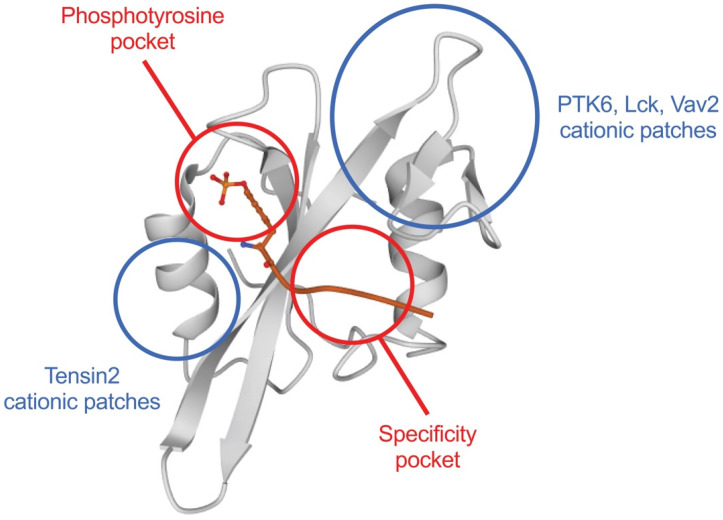
Visualization of the interacting zones of certain SH2 domains. The cationic patches responsible for lipid binding (blue) in different SH2 domains do not overlap with the pockets employed for specific phosphotyrosine recognition (red). For the generation of the schematic representation of the SH2 domain, we used the RCSB PDB database (www.rcsb.org accessed on 3 May 2021).

Recently, Park at al. published a study in which they thoroughly and systematically investigated the potential roles of lipids in regulating SH2 domain-mediated protein–protein interactions and cellular signaling activities [45]. Out of the 121 human SH2 domains, they successfully prepared and obtained 75 in amounts sufficient for biophysical studies, in which a primordial yeast SH2 domain was also included [45]. In surface plasmon resonance analyses, these SH2 domains were tested on lipid vesicles with a composition mimicking the cytosolic surface of the plasma membrane. Three-quarters of the SH2 domains bound the plasma membrane-mimetic vesicles with affinities comparable to that of established lipid-binding proteins: thirteen had K_d_ values in the micromolar range, and only eight SH2 domains displayed no detectable binding. Eighteen of the SH2 domains with high affinity for plasma membrane-mimetic vesicles were further examined to investigate the possible selectiveness towards different phosphoinositide species [45]. Among these, 12 showed phosphoinositide recognition bias compared with that of the Akt1/PKB PH domain, a canonical phosphoinositide-binding domain. Interestingly, these SH2 domains preferred phosphatidylinositol-4,5-bisphosphate or phosphatidylinositol-3,4,5-trisphosphate over other phosphoinositides. Three representative mCherry-tagged SH2 domains with different phosphoinositide selectivities were studied in vivo using fluorescent microscopy. Consistent with the in vitro data, all three were detected at the plasma membrane. Phosphatidylinositol-4,5-bisphosphate depletion of the plasma membrane significantly displaced the phosphatidylinositol-4,5-bisphosphate-selective and nonselective SH2 domains, but not the phosphatidylinositol-3,4,5-trisphosphate-selective one from the plasma membrane [45]. On the other hand, phosphatidylinositol-3,4,5-trisphosphate enrichment undoubtedly enhanced plasma membrane localization of the phosphatidylinositol-3,4,5-trisphosphate-selective SH2 domain in a specific manner (Figure 1). Phosphoinositide binding by SH2 domains seemed to be mediated by specific recognition of the lipid head-group because they clearly distinguished isoelectric but constitutionally different phosphatidylinositol-bisphosphates in vitro. Electrostatic potential computation showed that many human SH2 domains contain cationic patches near the phosphotyrosine binding pocket (Figure 2). These alternate cationic patches are less electropositive compared with the phosphotyrosine binding pockets, and nearly all of them are flanked by aromatic or hydrophobic amino acid side chains, a construction reminiscent of lipid-binding sites in membrane-binding proteins. Mutational analyses showed that instead of the phosphotyrosine binding pockets, the alternative cationic patches served as the primary binding sites for lipids in most SH2 domains. The location of the alternative cationic patch is highly variable within different SH2 domains. More importantly, the alternative cationic patch may form a pocket/groove or be presented on flat surfaces. Computational analyses suggested that the depth of the alternate cationic patch correlates with its specificity in lipid recognition. Nevertheless, these alternative cationic patches in SH2 domains are noticeably smaller structures compared with the lipid-binding sites of PH domains, suggesting the existence of multiple phosphoinositide recognition mechanisms (Figure 2) [45]. 

In the above referred work, Park at al. also exemplified the physiological importance of the lipid-binding activities of SH2 domains with PTK6 and ZAP70 [45]. The role of non-specific lipid binding by SH2 domains was illustrated with PTK6, a cytoplasmic tyrosine kinase that is not anchored to the plasma membrane [45]. This kinase is aberrantly expressed in certain human cancers [46]. Active PTK6 is tyrosine autophosphorylated and localized at the plasma membrane where it stimulates Erk5 and Akt/PKB [47]. According to in vitro experiments and predictive data, PTK6 contains an SH2 domain with a presumed non-specific binding site for anionic lipids. The researchers found that mutational destruction of this site reduced PTK6 plasma membrane affinity and localization, tyrosine autophosphorylation, and its ability to induce Erk5 and Akt/PKB phosphorylation.

ZAP70 is a Syk family tyrosine kinase with a central role in T cell antigen receptor signaling [48]. ZAP70 has tandem SH2 domains with which it binds to double tyrosine motifs of TCR-ζ ITAMs, upon which it tyrosine phosphorylates LAT and SLP-76 [49]. Modeling of the ZAP70 SH2 domains suggested that in the C-terminal SH2 domain, K176 and K186 could be involved in selective phosphatidylinositol-3,4,5-trisphosphate recognition, while K206 and K251 might mediate non-specific interactions with anionic membrane lipids. Mutational analyses showed that abrogation of the putative non-specific lipid-binding site resulted in decreased membrane affinity, unaltered phosphatidylinositol-3,4,5-trisphosphate preference, and suppressed signaling ability. On the other hand, elimination of the supposed phosphatidylinositol-3,4,5-trisphosphate-selective binding site decreased membrane affinity and abolished phosphatidylinositol-3,4,5-trisphosphate sensitivity, while it only reduced the signaling competence in the later minutes upon receptor activation. These results suggest that phosphatidylinositol-3,4,5-trisphosphate engagement via the SH2 domain is important for sustained ZAP70 activity but not for activation itself [45].

The SH2 domain of Lck, another key player in TCR signaling, has been dissected by Sheng et al. [50]. Lck is an Src-family protein tyrosine kinase that phosphorylates ITAMs in the CD3 and the ζ chain of the TCR-CD3 complex, thereby contributing to the initiation of TCR signaling [51,52]. The Lck SH2 domain was found to bind anionic lipids with high affinity, albeit with low specificity [50]. Electrostatic potential calculations, NMR analysis, and mutational studies identified a lipid-binding site in the Lck SH2 domain. It is an alternative cationic patch (distinct from the phosphotyrosine binding pocket) that includes K182 and R184 along with surface-exposed aromatic and hydrophobic residues. Mutation of the lipid-binding residues greatly reduced the interaction of Lck with the ζ chain in the activated TCR signaling complex and its overall TCR signaling activities. These results suggest that plasma membrane lipids, such as phosphatidylinositol-4,5-bisphosphate and phosphatidylinositol-3,4,5-trisphosphate, modulate the interaction of Lck with its binding partners in the TCR signaling complex and its TCR signaling activities in a spatiotemporally specific manner through its SH2 domain. In the proposed model, lipids generated by PI3K around the activated TCR-CD3 complex bind to the SH2 domain, thereby helping to maintain the open, i.e., active, conformation of Lck [50].

Based on the results of their previous systematic study on SH2 domain lipid binding, Kim et al. have investigated C1-Ten/Tensin2 [53]. This protein is highly similar in amino acid sequence and domain organization to Tensin [54]. In its C-terminal region, C1-Ten/Tensin2 contains an SH2 domain and a phosphotyrosine binding (PTB) domain, thus providing two potential interaction sites with phosphorylated tyrosine residues. There is also a conserved protein tyrosine phosphatase motif near the N terminus that shows significant structural homology to the tumor suppressor protein PTEN [55]. Wherever it is expressed, C1-Ten/Tensin2 is an accepted negative regulator of the Akt/PKB signal transduction pathway, and it inhibits cell survival, proliferation, and migration [56,57]. However, C1-Ten/Tensin2 seems to be a protein tyrosine, but not a lipid phosphatase, which was demonstrated on the insulin receptor substrate protein IRS-1 [58]. It preferentially dephosphorylates Y612 of IRS-1, which is associated with accelerated IRS-1 degradation. In their study, Kim et al. found that C1-Ten/Tensin2 attenuates IRS-1 phosphorylation in a PI3K-dependent manner [53]. Structural analyses of the C1-Ten/Tensin2 SH2 domain have suggested that it also possesses a distinct cationic patch formed by K1147, K1155, and K1157, which is far from the pY-binding pocket. In vitro mutational analyses have shown that this alternative cationic patch in the SH2 domain can mediate specific recognition of PI3K products, preferentially phosphatidylinositol-3,4,5-trisphosphate, but that it is not involved in interactions with tyrosine-phosphorylated peptides. Fluorescent microscopy studies with wild type and mutant C1-Ten/Tensin2 have not indicated that phosphatidylinositol-3,4,5-trisphosphate recognition by the alternative cationic patch of the SH2 domain could be involved in the plasma membrane targeting of the protein during insulin signaling. The alternative cationic patch has also not been found to be essential for the intrinsic phosphotyrosine phosphatase activity. On the other hand, it has indeed turned out to be important for the insulin-induced phosphotyrosine phosphatase activity of C1-Ten/Tensin2 towards IRS-1. These results show that the recognition of phosphatidylinositol-3,4,5-trisphosphate by the C1-Ten/Tensin2 SH2 domain is essential for the cellular signaling function of the protein. In addition, this finding supports a novel mechanism where the specific SH2 domain-phosphotyrosine recognition is facilitated by the lipid-binding ability of the SH2 domain [53,54,55,56,57,58].

One of the most recent studies on SH2 domain–lipid interactions focused on Vav2 [59], a ubiquitous guanine nucleotide exchange factor for Rho family GTPases that is involved in the regulation of a wide variety of biological processes [60,61]. Via its SH2 domain, Vav2 interacts with many different tyrosine-phosphorylated cell-surface receptors [62]. Ge et al. have reported that the Vav2 SH2 domain can specifically recognize phosphatidylinositol-4,5-bisphosphate and phosphatidylinositol-3,4,5-trisphosphate but binds them weakly [59]. The revealed lipid-binding site in Vav2-SH2 was found to be adjacent to its highly cationic phosphotyrosine binding pocket. However, NMR analysis suggested that the phosphotyrosine binding pocket might not be involved in lipid binding [59]. The lipid-binding site in the SH2 domain of Vav2 is quite similar to that of the Lck SH2 domain. While the Lck SH2 domain contains a key cationic residue (R184) that is involved in its lipid-binding activity, this position is replaced with a hydrophobic residue in Vav2-SH2, which might explain the weaker lipid-binding of Vav2-SH2. Although based solely on in vitro experiments, a physiological role for lipid binding by the Vav2-SH2 domain was also suggested here. Vav2 is known to convey signals from Eph-family receptors. The binding of Vav2 to EphA2 is crucial for EphA2-mediated tumor angiogenesis [63]. Experiments employing tyrosine-phosphorylated peptides derived from the juxtamembrane region of EphA2 showed that Vav-SH2-EphA2 recognition is independent of phosphatidylinositol-4,5-bisphosphate or phosphatidylinositol-3,4,5-trisphosphate binding [59]. The weak phosphatidylinositol-4,5-bisphosphate or phosphatidylinositol-3,4,5-trisphosphate binding ability of Vav2 thus may contribute to its membrane recruitment [59]. Recent molecular dynamic simulations revealed that the proximal juxtamembrane domain along with the EphA2 kinase domain interacts with phosphatidylinositol-4,5-bisphosphate and that both domains induce the formation of phosphatidylinositol-4,5-bisphosphate nanoclusters in the membrane [64,65]. Together, these findings highlight a complex functional cross-talk between EphA2, Vav2, and phosphatidylinositol-4,5-bisphosphate in vivo, which should be investigated further.

## 3. SH3 Domains Function from Constitutive through Regulated Protein Binding to Lipid Recognition

**Figure 3:**
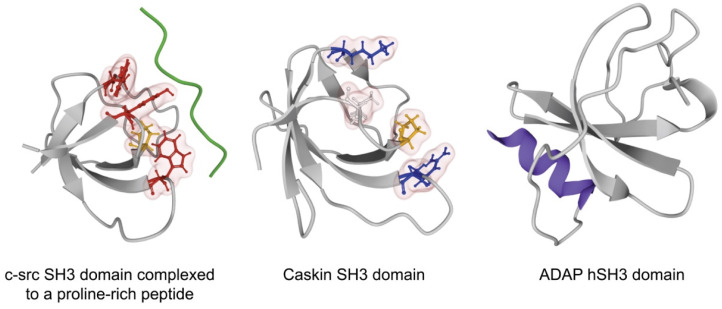
Comparison of lipid binding SH3 domains. The prototype c-src SH3 domain employs key aromatic (red) and proline (yellow) residues for binding proline-rich motifs of their partner (green). The Caskin SH3 domain traded off the three key aromatic residues for basic (blue) and hydrophobic (white) ones. The hSH3 domain of ADAP features an N-terminal helix (bue) rich in basic amino acids. We used the RCSB PDB database (www.rcsb.org accessed on 3 May 2021) for the source of SH3 domain structure representations.

Among the domains identified so far, the most abundant is the family of SH3 domains, which comprises more than 300 members nested in over 200 proteins in humans [16,66]. The host proteins are involved in diverse signaling pathways, including cell growth regulation, endocytosis, and cytoskeleton control. The canonical SH3 domain is a protein-interaction domain comprising 60 amino acids, consisting of five β-strands connected by loops (RT, N-Src, distal) and a short 3_10_ helix folded together into a β-sandwich of two antiparallel β-sheets [67]. SH3 domains fold to present a hydrophobic binding surface, the PXXP-binding site, adapted for the recognition of left handed proline-rich type II (PPII) helices [12,68]. The ligand-binding surface is relatively flat, and it consists of three shallow depressions, of which two are hydrophobic and defined by one pair of conserved aromatic residues each and one is formed by the RT and N-Src loops and possesses residues that add specificity (Figure 3) [69,70]. For example, in the human c-Src SH3 domain, Y93, Y139, W121, and P136 establish the two hydrophobic areas while D102 defines the specificity zone. In most canonical SH3 domains, the specificity zone is negatively charged and recognizes a positively charged residue located on either side of the PXXP motif, defining the ligand backbone orientation. The PPII helix has three residues per turn, making it practically triangular in cross-section; therefore, the base of this triangle sits on the ligand-binding surface of the SH3 domain. The two hydrophobic areas of the SH3 domain accommodate two hydrophobic-proline (ΦP) dipeptides in register on two adjacent turns of the recognized helix, whereas the specificity zone, in most cases, interacts with a basic residue in the ligand distal to the PXXP core. Due to the pseudo-symmetrical structure of the PPII helix, the PXXP-binding site can recognize proline-rich peptides in both orientations via two different binding modes. Based on peptide-binding preferences, two classes of canonical SH3 domains have been defined [71]. Class I SH3 domains recognize peptides that conform to the consensus motif RXXPXXP in a plus orientation. Class II domains bind to peptides that conform to the consensus PXXPXR in a minus orientation. On the other hand, a growing number of studies have revealed alternative recognition mechanisms [71].

Teyra et al. have comprehensively surveyed the specificity landscape of human SH3 domains in an unbiased manner via peptide-phage display and deep sequencing [72]. Based on approximately 70,000 unique binding peptides, they obtained 154 specificity profiles for 115 SH3 domains, revealing that roughly 50% of SH3 domains exhibit non-canonical specificities (devoid of the PXXP motif) and that they can recognize a wide variety of peptide motifs, most of which were previously unknown. Crystal structures of SH3 domains with two distinct non-canonical specificities revealed novel peptide-binding mechanisms that are mediated by an extended surface outside of the canonical proline-binding site [72].

Although the SH3 domain was originally accepted to mediate inter- and intramolecular protein–protein interactions in a constitutive manner, an accumulating body of evidence suggests more nuanced functions. The phosphorylation state of the tyrosine in the atypical binding motif RKXXY has been shown to regulate their binding interactions, suggesting that peptide recognition by the SH3 domain might be coupled to phosphotyrosine signaling [73]. On the other hand, phosphorylation at different tyrosine residues in SH3 domains themselves has been reported [74]. In several cases, the functional consequences have also been investigated. Merő et al. presented the first crystal structures of tyrosine-phosphorylated human SH3 domains derived from the Abelson-family kinases ABL1 and ABL2 [75]. The structures revealed that simultaneous phosphorylation of Y89 and Y134 in ABL1 or the homologous residues Y116 and Y161 in ABL2 induces only minor structural changes. Instead, the phosphate groups themselves sterically block the ligand-binding surface, thereby strongly inhibiting interactions with proline-rich peptide ligands. Extensive analysis of relevant literature and databases revealed not only that the residues phosphorylated in these model systems are well-conserved in other human SH3 domains, but also that the corresponding tyrosine residues are, in many cases, known phosphorylation sites in vivo. These results suggest that tyrosine phosphorylation of SH3 domains might be a mechanism involved in the regulation of the human SH3 interactome [75].

Interestingly, c-Src, the protein after which the SH domains were named, has been shown to directly bind acidic phospholipids on several sites of the protein [76,77]. While the N-terminal myristoylation site in the SH4 domain accounts for c-Src attachment to neutral membranes, the adjacent region, which contains a patch of basic residues, is responsible for attracting c-Src to phosphatidylserine-containing vesicles via electrostatic interaction. Recently, two additional independent lipid-binding sites have been identified in proximity to this region: one in the so called “Unique” motif and another one in the SH3 domain. The latter site includes residues in the RT and nSrc loops of the SH3 domain and is located on the opposite surface relative to the canonical PXXP-binding groove. Interestingly, the SH3 domain, with its stable fold, seems to act as an intramolecular scaffold for a complex of the SH4 domain and the intrinsically disordered “Unique” motif. The RT loop of the c-Src SH3 domain has been reported to also bind the myristoyl group of the SH4 domain when the protein is not anchored to a lipid membrane. Residues in the “Unique” region modulate this interaction. In the presence of liposomes or supported lipid bilayers, the myristoyl group is released to allow anchoring to the lipid bilayer, although the interaction of the SH4 and SH3 domains and the intramolecular complex is retained. Thus, the SH3 domain moves closer to the membrane surface and its orientation is restricted. The myristoyl group has been suggested to mediate c-Src dimerization via an interaction with the kinase domain. The mutation of amino acids in the kinase domain thought to be part of the myristoyl-binding site affected dimerization, supporting the existence of an additional myristoyl-binding site in the kinase domain. Thus, the myristoyl group can interact with the kinase domain of a second c-Src protein or the same c-Src protein via the SH3 domain, and these two binding events might be linked. These observations together link c-Src activation and membrane anchoring [76,77].

Analyses of some special SH3 domains have revealed that variants of the domain may mediate interactions with lipids rather than with protein sequences. (Table 1) Adhesion and degranulation-promoting adapter protein (ADAP, also known as FYB/SLAP-130) is critically involved in downstream signaling events prompted by activated T cell receptors [78,79]. T cell cytokine production, proliferation, and integrin clustering rely on ADAP function. A 70-residue stretch at the C-terminus of ADAP shows homology to SH3 domains; however, conserved residues are missing. Heuer et al. solved the three-dimensional structure of the ADAP C-terminal domain via NMR spectroscopy and demonstrated that it displays an altered SH3 domain fold [80]. In this case, an N-terminal, amphipathic helix makes extensive contacts to residues of the formal SH3 domain fold, thereby creating a composite surface with distinctive surface properties. This SH3 domain variant was proposed to be classified as a helically extended SH3 domain, or hSH3 domain for short (Figure 3). The ADAP C-terminal hSH3 domain is characterized by a lack of hydrophobic surface depressions. The ADAP hSH3 domain cannot bind conventional proline-rich peptides, and it is characterized by clusters of positively charged surface residues. Heuer et al. further scrutinized the structure and function of this domain [81]. Based on their findings related to these structural features, they investigated the possibility that ADAP hSH3 might interact with non-protein binding partners. The presence of a basic cluster comprising the α-helix and several residues within the SH3 scaffold guided their search for negatively charged molecules that can interact with the ADAP hSH3 domain. They observed that the hSH3 domain can bind to phospholipid membranes and that it has a preference for acidic phospholipids. A lipid overlay assay revealed that the hSH3 domain of ADAP can bind to several acidic lipids, including phosphatidylserine, phosphatidylinositol, phosphatidic acid, and polyphosphoinositides, but not to zwitterionic lipids such as phosphatidylcholine and phosphatidylethanolamine. A construct lacking the N-terminal helix did not exhibit any lipid-binding ability. The binding of the hSH3 domain to membranes containing acidic lipids was modeled via a multilamellar vesicle binding assay. While vesicles composed of a mixture of phosphatidylcholine and acidic lipids (phosphatidylserine and phosphoinositides) sedimented a substantial fraction of the protein, liposomes composed solely of zwitterionic phosphatidylcholine did not sediment hSH3. The results were confirmed via fluorescence resonance energy transfer assays with small, unilamellar vesicles. The affinity constants of hSH3 binding to phosphatidylcholine/phosphatidylserine and phosphatidylcholine/phosphatidylserine/phosphatidylinositol-4,5-bisphosphate vesicles were determined via an intrinsic tryptophan fluorescence-binding assay [81]. Sequence alignments of ADAP suggested that it contains an additional N-terminal hSH3 domain characterized by an amphipathic helix and a positively charged RT loop. 

PRAM-1 (promyelocytic-retinoic acid receptor alpha target gene encoding an adaptor molecule-1) is an ADAP homologue that also contains a fragment with the hallmarks of an hSH3 domain [82]. The properties of these three domains were compared [83]. Lipid overlay experiments showed that the ADAP N-terminal hSH3 and the PRAM-1 hSH3 domains can all bind to acidic phospholipids, while zwitterionic lipids such as phosphatidylcholine were not recognized. The strengths of the binding to vesicles containing acidic phospholipids followed the order ADAP hSH3 N-terminal < ADAP-hSH3 C-terminal < PRAM-1 hSH3. These domains all contain an amphipathic helix contacting the β-sheets of the canonical fold. It seems that while hydrophobic interactions are mainly responsible for the helix–sheet interaction, the surface-exposed amino acids of the helix are positively charged and are critical for efficient lipid binding. The RT loops of the lipid-binding hSH3 domains contain additional positively charged amino acids, and the number of net positive charge seems to correlate with the binding affinity of the hSH3 domains towards specific acidic phospholipids. The lipid-binding affinity observed for the hSH3 domain establishes it as low affinity module among the major class of lipid-binding domains, which includes the majority of PH domains. These domains are mostly insufficient to drive membrane localization alone, but are usually parts of larger membrane-proximal protein complexes that are anchored to the membrane via lipid modifications and additional protein–lipid interactions. It is possible that once ADAP is localized to the membrane, lipid binding by the hSH3 domain might alter the tertiary orientation of ADAP and its associated proteins. Functional investigations in Jurkat T cells with mutant ADAP indicated that the deletion of both amphipathic helices of the hSH3 domains reduces the ability of ADAP to enhance adhesion and migration in stimulated T cells [83].

**Figure 4:**
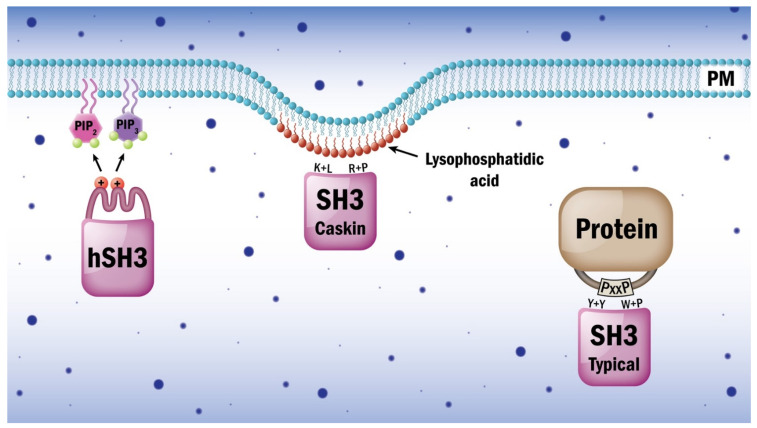
Some SH3 domains are involved in lipid recognition rather than protein binding. Typical SH3 domains accommodate two conserved pairs of aromatic/hydrophobic amino acids in two grooves for the attachment to two prolines in register (PXXP) on the surface of partner proteins. The helically extended SH3 (hSH3) domain identified in ADAP and PRAM-1 nests basic amino acids within an extra N-terminal helix. The composite surface created in the hSH3 domain displays cationic patches and can bind phosphatidylinositol-4,5-bisphosphate or phosphatidylinositol-3,4,5-trisphosphate but not proteins. The Caskin1 SH3 domain exchanged some proline-binding amino acids and lost the two PXXP motif-binding grooves. These changes resulted in a switch from protein to lipid binding ability. The Caskin1 SH3 domain preferentially binds to lysophosphatidic acid, a signaling-borne lipid found in positively charged membrane curvatures. Thus, hSH3 and Caskin1 SH3 domains might guide their host proteins along with their interacting partners to specific membrane sub-domains.

The calcium/calmodulin-dependent serine protein kinase (CASK)-interacting protein 1, or Caskin1, is enriched in neural synapses in mammals and regulates cortical actin filaments [84]. Based on its identified interaction partners and knock-out animal studies, Caskin1 might play various roles in neural function, and it is thought to participate in several pathological processes in the brain [85,86,87,88,89,90,91,92,93]. Caskin1 is a multidomain scaffold protein with a single, atypical SH3 domain in which key aromatic residues are missing from the canonical binding groove [94]. Despite the significance of the Caskin1 scaffold in synaptic information transfer, a physiologic protein partner for the SH3 domain of Caskin1 has not yet been identified. Koprivanacz et al. built a homology model of the SH3 domain of human Caskin1 and studied its biochemical properties in vitro [94]. Their model revealed that some of the key aromatic residues in the c-Src SH3 structure involved in ligand binding are substituted by basic or small hydrophobic residues in the Caskin1 SH3 domain (Figure 3). They hypothesized that this SH3 domain might be incompatible with the binding of proline-rich ligands but that lipids in the plasma membrane might serve as docking sites for the atypical SH3 domain of Caskin1 [94]. Signaling-related lysophospholipid mediators were implicated in this role. Intrinsic tryptophan fluorescence measurements were used to monitor lipid binding by this SH3 domain [94]. It was found that this domain selectively binds to oleoyl lysophosphatidic acid, whereas no binding was observed for the related lipid oleoyl lysophosphatidyl choline, which bears a phosphocholine head-group, as well as for sphingosine-1-phosphate and its precursor sphingosine [94]. Liposomes (as lipid carriers for lysophosphatidic acid) were used to further investigate the recognition of this lipid by the SH3 domain [94]. The interaction between the SH3 domain and lysophosphatidic acid turned out to be weak for monomeric lipid, but exhibits submicromolar affinity for lysophosphatidic acid-containing surfaces, either in micellar or in liposomal form [94]. Nuclear magnetic resonance experiments on the Caskin1 SH3 domain did not reveal any major structural changes upon lysophosphatidic acid addition but instead revealed a discrete set of amino acids that were affected by the binding (Figure 4). In their latest study, the research group presented a nuclear magnetic resonance-solved structure of the human Caskin1 SH3 domain, which validated the lack of a typical peptide binding groove [95]. Importantly, compared with the c-Src SH3 domain, the Y + Y and W + P pairs of the peptide binding pocket are replaced by K + L and R + P, respectively [95]. In addition, some of the negatively charged residues in the RT-loop are missing, while the distal-loop possesses an acidic region comprising D326 and D332 [95]. In the n-Src loop, a repositioning of acidic residues is found. Mapping of the amino acids involved in lysophosphatidic acid binding to the nuclear magnetic resonance structure of the human Caskin1 SH3 domain provided structural evidence that the proline-rich peptide binding groove was indeed missing and demonstrated that lysophosphatidic acid binding involved a domain region distinct from the peptide binding groove generally shared by SH3 domains [95].

The membrane-born lipid mediator lysophosphatidic acid elicits many physiologic responses by acting on cognate G protein-coupled receptors as well as on intracellular target proteins [96]. Lysophospholipids influence the shape of membranes, and lysophosphatidic acid is the most potent inducer of positive membrane curvature [97]. Based on these facts and in vitro data on Caskin1, it might be speculated that the human Caskin1 SH3 domain is a novel lipid-binding domain with preference for the positive membrane curvatures generated by lysophosphatidic acid, supporting a yet unrecognized function of lysophosphatidic acid in intracellular signaling processes [94].

## 4. Perspectives

The SH2 domain was originally defined as a reader module in signal transduction specialized for the recognition of newly formed phosphotyrosine sites; however, many different pathways employ this matching pair of moieties. Accumulating evidence suggests that additional lipid-binding abilities that vary in distinct SH2 domains nested in individual proteins contribute to specificity in the precise deciphering and translation of cellular information held in altered membrane lipid composition. In this manner, the composition and assembly–disassembly dynamics of a signaling hub formed around a freshly arisen phosphotyrosine are also influenced by lipid second messengers via the SH2 domain. 

The SH3 domain is widely accepted as a protein domain that provides stable interactions for the formation of constitutive signaling sub-complexes. However, a handful of SH3 domains have been reported to specialize in lipid recognition instead of protein binding. With little experimental data, it remains to be elucidated whether the lipid-binding ability of SH3 domains is a recent innovation in domain evolution that recycles a structure for extremely specialized functions in niche roles or if it is an as yet overlooked general potential of the SH3 domain.

Additional specific biological responses might be controlled by the lipid-binding properties of some SH2 and SH3 domains, which could support spatiotemporal selection of the appropriate responding signaling protein from the repertoire of possible proteins for a given trigger. Experimental data are needed to address the questions stemming from the observations reviewed here. 

## Figures and Tables

**Table 1:** Overview of the non-conventional, lipid-binding SH2 and SH3 domain-containing proteins discussed throughout the text.

**SH2 Domains with Abilities to Bind Lipids in Addition to Phosphorylated Tyrosine Motifs**			
protein name	domain	specificity	biological relevance
**Abl**—non-receptor tyrosine kinase; proto-oncogene	SH2 domain	PIP_2_ interaction	mutually exclusive lipid or phosphotyrosine binding
**PTK6**—protein tyrosine kinas-6; EGFR signaling member	SH2 domain	binding site for anionic lipids	activation
**ZAP70**—Syk-related tyrosine kinase; T lymphocyte activation	C-terminal SH2 domain	PIP_3_ recognition, interactions with anionic membrane lipids	sustained activation
**Lck**—lymphocyte-specific protein tyrosine kinase; key player in initiation of TCR signaling	SH2 domain	binding of anionic lipids	sustained activation
**C1-Ten/Tensin2**—protein tyrosine phosphatase; negative regulator of the Akt/PKB signaling	C-terminal SH2 domain	preferential binding of PIP_3_	activation and specific targeting on IRS-1
**Vav2**—guanine nucleotide exchange factor for Rho family GTPases	SH2 domain	weak PIP_2_ and PIP_3_ interaction	targeting to membrane subdomains
**Atypical SH3 Domains without Recognition Motifs for Canonical Proline-Rich Structures**			
protein name	domain	specificity	biological relevance
**ADAP**—Adhesion and degranulation-promoting adapter protein	N-terminal hSH3 domain	binding to acidic phospholipids	
	C-terminal hSH3 domain	lipid-binding	specific orientation within a membrane-proximal protein complex
**PRAM-1**—promyelocytic-retinoic acid receptor alpha target gene encoding an adaptor molecule-1	hSH3 domain	acidic phospholipid binding	specific orientation within a membrane-proximal protein complex
**Caskin1**—calcium/calmodulin-dependent serine protein kinase-interacting protein 1; regulates neural synapses	atypical SH3 domain	binding to lysophospholipid mediators, especially to lysophosphatidic acid	targeting to membrane subdomains

## References

[1] Mayer B.J. (2015). The discovery of modular binding domains: Building blocks of cell signalling. Nat. Rev. Mol. Cell Biol..

[2] Sadowski I., Stone J.C., Pawson T. (1986). A noncatalytic domain conserved among cytoplasmic protein-tyrosine kinases modifies the kinase function and transforming activity of Fujinami sarcoma virus P130gag-fps. Mol. Cell. Biol..

[3] Anderson D., Koch C.A., Grey L., Ellis C., Moran M.F., Pawson T. (1990). Binding of SH2 domains of phospholipase Cγ1, GAP, and Src to activated growth factor receptors. Science.

[4] Margolis B., Li N., Koch A., Mohammadi M., Hurwitz D.R., Zilberstein A., Ullrich A., Pawson T., Schlessinger J. (1990). The tyrosine phosphorylated carboxyterminus of the EGF receptor is a binding site for GAP and PLC-γ. EMBO J..

[5] Matsuda M., Mayer B.J., Hanafusa H. (1991). Identification of domains of the v-crk oncogene product sufficient for association with phosphotyrosine-containing proteins. Mol. Cell. Biol..

[6] Mayer B.J., Jackson P.K., Baltimore D. (1991). The noncatalytic src homology region 2 segment of abl tyrosine kinase binds to tyrosine-phosphorylated cellular proteins with high affinity. Proc. Natl. Acad. Sci. USA.

[7] Moran M.F., Koch C.A., Anderson D., Ellis C., England L., Martin G.S., Pawson T. (1990). Src homology region 2 domains direct protein-protein interactions in signal transduction. Proc. Natl. Acad. Sci. USA.

[8] Mayer B.J., Hamaguchi M., Hanafusa H. (1988). A novel viral oncogene with structural similarity to phospholipase C. Nature.

[9] Stahl M.L., Ferenz C.R., Kelleher K.L., Kriz R.W., Knopf J.L. (1988). Sequence similarity of phospholipase C with the non-catalytic region of src. Nature.

[10] Kurochkina N., Guha U. (2013). SH3 domains: Modules of protein-protein interactions. Biophys. Rev..

[11] Cicchetti P., Mayer B.J., Thiel G., Baltimore D. (1992). Identification of a protein that binds to the SH3 region of abl and is similar to Bcr and GAP-rho. Science.

[12] Ren R., Mayer B.J., Cicchetti P., Baltimore D. (1993). Identification of a ten-amino acid proline-rich SH3 binding site. Science.

[13] Kay B.K., Williamson M.P., Sudol M. (2000). The importance of being proline: The interaction of proline-rich motifs in signaling proteins with their cognate domains. FASEB J..

[14] Zarrinpar A., Bhattacharyya R.P., Lim W.A. (2003). The structure and function of proline recognition domains. Sci. STKE.

[15] Wunderlich L., Gohér Á., Faragó A., Downward J., Buday L. (1999). Requirement of multiple SH3 domains of Nck for ligand binding. Cell. Signal..

[16] Kärkkäinen S., Hiipakka M., Wang J.-H., Kleino I., Vähä-Jaakkola M., Renkema G.H., Liss M., Wagner R., Saksela K. (2006). Identification of preferred protein interactions by phage-display of the human Src homology-3 proteome. EMBO Rep..

[17] Stahelin R.V. (2009). Lipid binding domains: More than simple lipid effectors. J. Lipid Res..

[18] Takai Y., Kishimoto A., Iwasa Y., Kawahara Y., Mori T., Nishizuka Y. (1979). Calcium-dependent activation of a multifunctional protein kinase by membrane phospholipids. J. Biol. Chem..

[19] Zhang G., Kazanietz M.G., Blumberg P.M., Hurley J.H. (1995). Crystal structure of the Cys2 activator-binding domain of protein kinase Cδ in complex with phorbol ester. Cell.

[20] Sutton R.B., Davletov B.A., Berghuis A.M., Sudhof T.C., Sprang S.R. (1995). Structure of the first C2 domain of synaptotagmin I: A novel Ca^2+^/phospholipid-binding fold. Cell.

[21] Ferguson K.M., Lemmon M.A., Schlessinger J., Sigler P.B. (1995). Structure of the high affinity complex of inositol trisphosphate with a phospholipase C pleckstrin homology domain. Cell.

[22] Corradi V., Sejdiu B.I., Mesa-Galloso H., Abdizadeh H., Noskov S.Y., Marrink S.J., Tieleman D.P. (2019). Emerging Diversity in Lipid-Protein Interactions. Chem. Rev..

[23] Pawson T. (2004). Specificity in Signal Transduction: From Phosphotyrosine-SH2 Domain Interactions to Complex Cellular Systems. Cell.

[24] Waksman G., Shoelson S.E., Pant N., Cowburn D., Kuriyan J. (1993). Binding of a high affinity phosphotyrosyl peptide to the Src SH2 domain: Crystal structures of the complexed and peptide-free forms. Cell.

[25] Ladbury J.E., Arold S. (2000). Searching for specificity in SH domains. Chem. Biol..

[26] Machida K., Mayer B.J. (2005). The SH2 domain: Versatile signaling module and pharmaceutical target. Biochim. Biophys. Acta Proteins Proteom..

[27] Good M.C., Zalatan J.G., Lim W.A. (2011). Scaffold proteins: Hubs for controlling the flow of cellular information. Science.

[28] Scott J.D., Pawson T. (2009). Cell signaling in space and time: Where proteins come together and when they’re apart. Science.

[29] Bray D. (1998). Signaling complexes: Biophysical constraints on intracellular communication. Annu. Rev. Biophys. Biomol. Struct..

[30] Cho W. (2006). Building signaling complexes at the membrane. Sci. STKE.

[31] Bae J.H., Lew E.D., Yuzawa S., Tomé F., Lax I., Schlessinger J. (2009). The Selectivity of Receptor Tyrosine Kinase Signaling Is Controlled by a Secondary SH2 Domain Binding Site. Cell.

[32] Chen Y., Sheng R., Källberg M., Silkov A., Tun M.P., Bhardwaj N., Kurilova S., Hall R.A., Honig B., Lu H. (2012). Genome-wide Functional Annotation of Dual-Specificity Protein- and Lipid-Binding Modules that Regulate Protein Interactions. Mol. Cell.

[33] Feng W., Zhang M. (2009). Organization and dynamics of PDZ-domain-related supramodules in the postsynaptic density. Nat. Rev. Neurosci..

[34] Sheng R., Chen Y., Yung Gee H., Stec E., Melowic H.R., Blatner N.R., Tun M.P., Kim Y., Källberg M., Fujiwara T.K. (2012). Cholesterol modulates cell signaling and protein networking by specifically interacting with PDZ domain-containing scaffold proteins. Nat. Commun..

[35] Sheng R., Kim H., Lee H., Xin Y., Chen Y., Tian W., Cui Y., Choi J.C., Doh J., Han J.K. (2014). Cholesterol selectively activates canonical Wnt signalling over non-canonical Wnt signalling. Nat. Commun..

[36] Wu H., Feng W., Chen J., Chan L.N., Huang S., Zhang M. (2007). PDZ Domains of Par-3 as Potential Phosphoinositide Signaling Integrators. Mol. Cell.

[37] Zimmermann P., Meerschaert K., Reekmans G., Leenaerts I., Small J.V., Vandekerckhove J., David G., Gettemans J. (2002). PIP2-PDZ domain binding controls the association of syntenin with the plasma membrane. Mol. Cell.

[38] Ravichandran K.S., Zhou M.M., Pratt J.C., Harlan J.E., Walk S.F., Fesik S.W., Burakoff S.J. (1997). Evidence for a requirement for both phospholipid and phosphotyrosine binding via the Shc phosphotyrosine-binding domain in vivo. Mol. Cell. Biol..

[39] Rameh L.E., Chen C.S., Cantley L.C. (1995). Phosphatidylinositol (3,4,5)P3 interacts with SH2 domains and modulates PI 3-kinase association with tyrosine-phosphorylated proteins. Cell.

[40] Bae Y.S., Cantley L.G., Chen C.S., Kim S.R., Kwon K.S., Rhee S.G. (1998). Activation of phospholipase C-γ by phosphatidylinositol 3,4,5- trisphosphate. J. Biol. Chem..

[41] Lo Surdo P., Bottomley M.J., Arcaro A., Siegal G., Panayotou G., Sankar A., Gaffney P.R.J., Riley A.M., Potter B.V.L., Waterfield M.D. (1999). Structural and biochemical evaluation of the interaction of the phosphatidylinositol 3-kinase p85α Src homology 2 domains with phosphoinositides and inositol polyphosphates. J. Biol. Chem..

[42] Tokonzaba E., Capelluto D.G.S., Kutateladze T.G., Overduin M. (2006). Phosphoinositide, phosphopeptide and pyridone interactions of the ABL SH2 domain. Chem. Biol. Drug Des..

[43] Bradley W.D., Koleske A.J. (2009). Regulation of cell migration and morphogenesis by Abl-family kinases: Emerging mechanisms and physiological contexts. J. Cell Sci..

[44] Greuber E.K., Smith-Pearson P., Wang J., Pendergast A.M. (2013). Role of ABL family kinases in cancer: From leukaemia to solid tumours. Nat. Rev. Cancer.

[45] Park M.J., Sheng R., Silkov A., Jung D.J., Wang Z.G., Xin Y., Kim H., Thiagarajan-Rosenkranz P., Song S., Yoon Y. (2016). SH2 Domains Serve as Lipid-Binding Modules for pTyr-Signaling Proteins. Mol. Cell.

[46] Brauer P.M., Tyner A.L. (2009). RAKing in AKT: A tumor suppressor function for the intracellular tyrosine kinase FRK. Cell Cycle.

[47] Zheng Y., Asara J.M., Tyner A.L. (2012). Protein-tyrosine kinase 6 promotes peripheral adhesion complex formation and cell migration by phosphorylating p130 CRK-associated substrate. J. Biol. Chem..

[48] Wang H., Kadlecek T.A., Au-Yeung B.B., Goodfellow H.E.S., Hsu L.Y., Freedman T.S., Weiss A. (2010). ZAP-70: An essential kinase in T-cell signaling. Cold Spring Harb. Perspect. Biol..

[49] Pollizzi K.N., Powell J.D. (2014). Integrating canonical and metabolic signalling programmes in the regulation of T cell responses. Nat. Rev. Immunol..

[50] Sheng R., Jung D.J., Silkov A., Kim H., Singaram I., Wang Z.G., Xin Y., Kim E., Park M.J., Thiagarajan-Rosenkranz P. (2016). Lipids regulate Lck protein activity through their interactions with the Lck Src homology 2 domain. J. Biol. Chem..

[51] Palacios E.H., Weiss A. (2004). Function of the Src-family kinases, Lck and Fyn, in T-cell development and activation. Oncogene.

[52] Salmond R.J., Filby A., Qureshi I., Caserta S., Zamoyska R. (2009). T-cell receptor proximal signaling via the Src-family kinases, Lck and Fyn, influences T-cell activation, differentiation, and tolerance. Immunol. Rev..

[53] Kim E., Kim D.H., Singaram I., Jeong H., Koh A., Lee J., Cho W., Ryu S.H. (2018). Cellular phosphatase activity of C1-Ten/Tensin2 is controlled by Phosphatidylinositol-3,4,5-triphosphate binding through the C1-Ten/Tensin2 SH2 domain. Cell. Signal..

[54] Hafizi S., Alindri F., Karlsson R., Dahlbäck B. (2002). Interaction of Axl receptor tyrosine kinase with C1-TEN, a novel C1 domain-containing protein with homology to tensin. Biochem. Biophys. Res. Commun..

[55] Kim J.H., Liao D., Lau L.F., Huganir R.L. (1998). SynGAP: A synaptic RasGAP that associates with the PSD-95/SAP90 protein family. Neuron.

[56] Hafizi S., Ibraimi F., Dahlbäck B. (2005). C1-TEN is a negative regulator of the Akt/PKB signal transduction pathway and inhibits cell survival, proliferation, and migration. FASEB J..

[57] Hafizi S., Gustafsson A., Oslakovic C., Idevall-Hagren O., Tengholm A., Sperandio O., Villoutreix B.O., Dahlbäck B. (2010). Tensin2 reduces intracellular phosphatidylinositol 3,4,5-trisphosphate levels at the plasma membrane. Biochem. Biophys. Res. Commun..

[58] Koh A., Lee M.N., Yang Y.R., Jeong H., Ghim J., Noh J., Kim J., Ryu D., Park S., Song P. (2013). C1-Ten Is a Protein Tyrosine Phosphatase of Insulin Receptor Substrate 1 (IRS-1), Regulating IRS-1 Stability and Muscle Atrophy. Mol. Cell. Biol..

[59] Ge L., Wu B., Zhang Y., Wang J., Zhao H., Wang J. (2020). Biochemical and NMR characterization of the interactions of Vav2-SH2 domain with lipids and the EphA2 juxtamembrane region on membrane. Biochem. J..

[60] Tamás P., Solti Z., Bauer P., Illés A., Sipeki S., Bauer A., Faragó A., Downward J., Buday L. (2003). Mechanism of epidermal growth factor regulation of Vav2, a guanine nucleotide exchange factor for Rac. J. Biol. Chem..

[61] Bustelo X.R. (2014). Vav family exchange factors: An integrated regulatory and functional view. Small GTPases.

[62] Bustelo X.R. (2001). Vav proteins, adaptors and cell signaling. Oncogene.

[63] Hunter S.G., Zhuang G., Brantley-Sieders D., Swat W., Cowan C.W., Chen J. (2006). Essential Role of Vav Family Guanine Nucleotide Exchange Factors in EphA Receptor-Mediated Angiogenesis. Mol. Cell. Biol..

[64] Chavent M., Karia D., Kalli A.C., Domański J., Duncan A.L., Hedger G., Stansfeld P.J., Seiradake E., Jones E.Y., Sansom M.S.P. (2018). Interactions of the EphA2 Kinase Domain with PIPs in Membranes: Implications for Receptor Function. Structure.

[65] Hedger G., Sansom M.S.P., Koldsø H. (2015). The juxtamembrane regions of human receptor tyrosine kinases exhibit conserved interaction sites with anionic lipids. Sci. Rep..

[66] Pawson T., Schlessingert J. (1993). SH2 and SH3 domains. Curr. Biol..

[67] Musacchio A., Gibson T., Lehto V.P., Saraste M. (1992). SH3—An abundant protein domain in search of a function. FEBS Lett..

[68] Yu H., Chen J.K., Feng S., Dalgarno D.C., Brauer A.W., Schrelber S.L. (1994). Structural basis for the binding of proline-rich peptides to SH3 domains. Cell.

[69] Saksela K., Permi P. (2012). SH3 domain ligand binding: What’s the consensus and where’s the specificity?. FEBS Lett..

[70] Lim W.A., Richards F.M., Fox R.O. (1994). Structural determinants of peptide-binding orientation and of sequence specificity in SH3 domains. Nature.

[71] Feng S., Chen J.K., Yu H., Simon J.A., Schreiber S.L. (1994). Two binding orientations for peptides to the Src SH3 domain: Development of a general model for SH3-ligand interactions. Science.

[72] Teyra J., Huang H., Jain S., Guan X., Dong A., Liu Y., Tempel W., Min J., Tong Y., Kim P.M. (2017). Comprehensive Analysis of the Human SH3 Domain Family Reveals a Wide Variety of Non-canonical Specificities. Structure.

[73] Kang H. (2000). SH3 domain recognition of a proline-independent tyrosine-based RKxxYxxY motif in immune cell adaptor SKAP55. EMBO J..

[74] Tatárová Z., Brábek J., Rösel D., Novotný M. (2012). SH3 domain tyrosine phosphorylation—Sites, role and evolution. PLoS ONE.

[75] Mero B., Radnai L., Gógl G., Tőke O., Leveles I., Koprivanacz K., Szeder B., Dülk M., Kudlik G., Virág Vas X. (2019). Structural insights into the tyrosine phosphorylation–mediated inhibition of SH3 domain–ligand interactions. J. Biol. Chem..

[76] Pérez Y., Maffei M., Igea A., Amata I., Gairí M., Nebreda A.R., Bernadó P., Pons M. (2013). Lipid binding by the Unique and SH3 domains of c-Src suggests a new regulatory mechanism. Sci. Rep..

[77] Le Roux A.L., Mohammad I.L., Mateos B., Arbesú M., Gairí M., Khan F.A., Teixeira J.M.C., Pons M. (2019). A Myristoyl-Binding Site in the SH3 Domain Modulates c-Src Membrane Anchoring. iScience.

[78] Da Silva A.J., Li Z., De Vera C., Canto E., Findell P., Rudd C.E. (1997). Cloning of a novel T-cell protein FYB that binds FYN and SH2-domain-containing leukocyte protein 76 and modulates interleukin 2 production. Proc. Natl. Acad. Sci. USA.

[79] Musci M.A., Hendricks-Taylor L.R., Motto D.G., Paskind M., Kamens J., Turck C.W., Koretzky G.A. (1997). Molecular cloning of SLAP-130, an SLP-76-associated substrate of the T cell antigen receptor-stimulated protein tyrosine kinases. J. Biol. Chem..

[80] Heuer K., Kofler M., Langdon G., Thiemke K., Freund C. (2004). Structure of a helically extended SH3 domain of the T cell adapter protein ADAP. Structure.

[81] Heuer K., Arbuzova A., Strauss H., Kofler M., Freund C. (2005). The helically extended SH3 domain of the T cell adaptor protein ADAP is a novel lipid interaction domain. J. Mol. Biol..

[82] Moog-Lutz C., Peterson E.J., Lutz P.G., Eliason S., Cavé-Riant F., Singer A., Di Gioia Y., Dmowski S., Kamens J., Cayre Y.E. (2001). PRAM-1 Is a Novel Adaptor Protein Regulated by Retinoic Acid (RA) and Promyelocytic Leukemia (PML)-RA Receptor α in Acute Promyelocytic Leukemia Cells. J. Biol. Chem..

[83] Heuer K., Sylvester M., Kliche S., Pusch R., Thiemke K., Schraven B., Freund C. (2006). Lipid-binding hSH3 Domains in Immune Cell Adapter Proteins. J. Mol. Biol..

[84] Tabuchi K., Biederer T., Butz S., Sudhof T.C. (2002). CASK participates in alternative tripartite complexes in which Mint 1 competes for binding with caskin 1, a novel CASK-binding protein. J. Neurosci..

[85] Daimon C.M., Jasien J.M., Wood W.H., Zhang Y., Becker K.G., Silverman J.L., Crawley J.N., Martin B., Maudsley S. (2015). Hippocampal transcriptomic and proteomic alterations in the BTBR mouse model of autism spectrum disorder. Front. Physiol..

[86] Lv X., Zhao K., Lan Y., Li Z., Ding N., Su J., Lu H., Song D., Gao F., He W. (2017). miR-21a-5p contributes to porcine hemagglutinating encephalomyelitis virus proliferation via targeting CASK-interactive protein1 in vivo and vitro. Front. Microbiol..

[87] Datta A., Jingru Q., Khor T.H., Teo M.T., Heese K., Sze S.K. (2011). Quantitative neuroproteomics of an in vivo rodent model of focal cerebral Ischemia/reperfusion injury reveals a temporal regulation of novel pathophysiological molecular markers. J. Proteome Res..

[88] Middleton F.A., Carrierfenster K., Mooney S.M., Youngentob S.L. (2009). Gestational ethanol exposure alters the behavioral response to ethanol odor and the expression of neurotransmission genes in the olfactory bulb of adolescent rats. Brain Res..

[89] Crockett D.K., Lin Z., Elenitoba-Johnson K.S.J., Lim M.S. (2004). Identification of NPM-ALK interacting proteins by tandem mass spectrometry. Oncogene.

[90] Katano T., Takao K., Abe M., Yamazaki M., Watanabe M., Miyakawa T., Sakimura K., Ito S. (2018). Distribution of Caskin1 protein and phenotypic characterization of its knockout mice using a comprehensive behavioral test battery. Mol. Brain.

[91] Bencsik N., Pusztai S., Borbély S., Fekete A., Dülk M., Kis V., Pesti S., Vas V., Szűcs A., Buday L. (2019). Dendritic spine morphology and memory formation depend on postsynaptic Caskin proteins. Sci. Rep..

[92] Oyazato Y., Iijima K., Emi M., Sekine T., Kamei K., Takanashi J., Nakao H., Namai Y., Nozu K. (2011). Molecular Analysis of TSC2/PKD1 Contiguous Gene Deletion Syndrome. Kobe J. Med. Sci..

[93] Boehm D., Bacher J., Neumann H.P.H. (2007). Gross Genomic Rearrangement Involving the TSC2-PKD1 Contiguous Deletion Syndrome: Characterization of the Deletion Event by Quantitative Polymerase Chain Reaction Deletion Assay. Am. J. Kidney Dis..

[94] Koprivanacz K., Tőke O., Besztercei B., Juhász T., Radnai L., Merő B., Mihály J., Péter M., Balogh G., Vígh L. (2017). The SH3 domain of Caskin1 binds to lysophosphatidic acid suggesting a direct role for the lipid in intracellular signaling. Cell. Signal..

[95] Tőke O., Koprivanacz K., Radnai L., Merő B., Juhász T., Liliom K., Buday L. (2021). Solution NMR Structure of the SH3 Domain of Human Caskin1 Validates the Lack of a Typical Peptide Binding Groove and Supports a Role in Lipid Mediator Binding. Cells.

[96] Lin M.E., Herr D.R., Chun J. (2010). Lysophosphatidic acid (LPA) receptors: Signaling properties and disease relevance. Prostaglandins Other Lipid Mediat..

[97] Kooijman E.E., Chupin V., de Kruijff B., Burger K.N.J. (2003). Modulation of membrane curvature by phosphatidic acid and lysophosphatidic acid. Traffic.

